# Distribution and characterization of ampicillin- and tetracycline-resistant *Escherichia coli *from feedlot cattle fed subtherapeutic antimicrobials

**DOI:** 10.1186/1471-2180-11-78

**Published:** 2011-04-19

**Authors:** Parasto Mirzaagha, Marie Louie, Ranjana Sharma, L Jay Yanke, Ed Topp, Tim A McAllister

**Affiliations:** 1 Agriculture and Agri-Food Canada, Lethbridge Research Centre, Lethbridge, Alberta, T1J 4B1, Canada; 2 Faculty of Medicine, University of Calgary, Calgary, Alberta, T2N 4N1, Canada; 3 Agriculture and Agri-Food Canada, Southern Crop Protection and Food Research Centre, London, Ontario, N5V 4T3, Canada

## Abstract

**Background:**

Feedlot cattle in North America are routinely fed subtherapeutic levels of antimicrobials to prevent disease and improve the efficiency of growth. This practice has been shown to promote antimicrobial resistance (AMR) in subpopulations of intestinal microflora including *Escherichia coli*. To date, studies of AMR in feedlot production settings have rarely employed selective isolation, therefore yielding too few AMR isolates to enable characterization of the emergence and nature of AMR in *E. coli *as an indicator bacterium. *E. coli *isolates (*n *= 531) were recovered from 140 cattle that were housed (10 animals/pen) in 14 pens and received no dietary antimicrobials (control - 5 pens, CON), or were intermittently administered subtherapeutic levels of chlortetracycline (5 pens-T), chlortetracycline + sulfamethazine (4 pens-TS), or virginiamycin (5 pens-V) for two separate periods over a 9-month feeding period. Phenotype and genotype of the isolates were determined by susceptibility testing and pulsed field gel electrophoresis and distribution of characterized isolates among housed cattle reported. It was hypothesized that the feeding of subtherapeutic antibiotics would increase the isolation of distinct genotypes of AMR *E. coli *from cattle.

**Results:**

Overall, patterns of antimicrobial resistance expressed by *E. coli *isolates did not change among diet groups (CON vs. antibiotic treatments), however; isolates obtained on selective plates (i.e., M^A^,M^T^), exhibited multi-resistance to sulfamethoxazole and chloramphenicol more frequently when obtained from TS-fed steers than from other treatments. Antibiograms and PFGE patterns suggested that AMR *E. coli *were readily transferred among steers within pens. Most M^T ^isolates possessed the *tet*(B) efflux gene (58.2, 53.5, 40.8, and 50.6% of isolates from CON, T, TS, and V steers, respectively) whereas among the M^A ^(ampicillin-resistant) isolates, the *tem1*-like determinant was predominant (occurring in 50, 66.7, 80.3, and 100% of isolates from CON, T, TS, and V steers, respectively).

**Conclusions:**

Factors other than, or in addition to subtherapeutic administration of antibiotics influence the establishment and transmission of AMR *E. coli *among feedlot cattle.

## Background

In North America, antimicrobials are often fed to feedlot cattle at subtherapeutic levels for disease prevention and to improve feed efficiency [1]. Although such a practice reduces production costs, it may also promote the development of antimicrobial resistance (AMR) both in pathogenic and in non-pathogenic bacteria [2]. It has been hypothesized that continuous, low-dose administration of antimicrobials increases the risk of AMR development, in comparison with short term, high-dose therapeutic use [3,4]. Concern also exists that subtherapeutic administration of antimicrobials promotes horizontal gene transfer between commensal and pathogenic bacteria [5].

In Canada, antimicrobials used for growth enhancement in livestock are approved through the guidelines established by the Food and Drugs Act and Regulations of Health Canada. Examples of antimicrobials presently approved for in-feed administration include tetracyclines, virginiamycin, penicillin, monensin, sulfonamides and tylosin. The potential risk to human health via promotion of AMR is perhaps greatest for those products used to treat both livestock and humans (i.e., tetracyclines and sulfonamides). There is also a concern that veterinary antimicrobials classed in the same antibiotic family as those used in human therapy may promote the development of cross-resistance. For example, the subtherapeutic use in livestock of virginiamycin, a streptogramin, may lead to resistance to Synercid^®^, an antibiotic of the same family, used as a last resort treatment of vancomycin-resistant *Enterococcus faecium *in humans [6].

Several studies (reviewed by [2]) have investigated the effect of administering subtherapeutic antimicrobials to swine and poultry on antibiotic resistance in commensal and pathogenic gut microflora, but comparatively few have examined the impact of this management practice on AMR in beef cattle [7,8]. Comparisons of organic and conventional livestock production systems [9], dairies [10] and of ground beef originating from conventional vs. "natural" sources [11] have generally revealed a higher prevalence of AMR in conventional systems. The majority of the studies that have been conducted are of an epidemiological nature and detailed characterization of the limited number of AMR isolates collected has not been undertaken.

Our research team recently conducted a comprehensive study to document the prevalence of AMR *Escherichia coli *among feedlot cattle being fed various antibiotics at subtherapeutic levels, in two intermittent periods, over the course of their growing and fattening periods [12]. From those data, we concluded that withdrawal of subtherapeutic antibiotics during the feeding period had little impact on the prevalence of tetracycline- or ampicillin-resistant *E. coli *in the cattle. In this paper, we present a more comprehensive assessment of 531 selected *E. coli *isolates collected from individual steers on four representative sampling days throughout the feeding period. Through phenotypic and genotypic characterization, the objective of this study was to explore the distribution of AMR *E. coli *among individual animals fed the different diets within the feedlot environment. It was hypothesized that the subtherapeutic administration of antibiotics would alter the occurrence of AMR *E. coli *phenotypes among animals.

## Methods

The *E. coli *isolates investigated in the present work were a sub-set of those archived during a larger study [12] in which prevalence of AMR *E. coli *was assessed over the course of a backgrounding/finishing feeding trial in a research feedlot. Full methodological detail of their isolation has been described previously [12], and is described briefly below.

### Animals, housing and diets

The study was conducted at the Lethbridge Research Centre feedlot (Lethbridge, Alberta, Canada) using crossbred steer calves penned in groups of 10. Cattle were housed in rows of parallel pens with the same antibiotic treatment administered to 5 adjacent pens. Pens were separated by porosity fencing and a pen-specific feed bunk lined the front of each pen. The bunk was of a sufficient length so that all individuals within a pen could feed at the same time. Cattle were retained in the pen throughout the feeding period and there was no need for equipment to enter any of the pens during the feeding period. Adjacent pens within each treatment shared a common water bowl, but the assignment of treatments to pens ensured that water bowls were shared only by steers in the same treatment group. Cattle were processed through a common handling area, but handled in the order of the control group first followed by the virginiamycin group, chlortetracycline group and finally the chlorotetracycline-sulfamethazine group (see below). The area was thoroughly cleaned after each group passed through the handling area. The calves used in the study received no antibiotics prior to or during shipment to the Lethbridge Research Centre feedlot. Furthermore, no subtherapeutic or therapeutic antibiotics were administered prior to this start of this study. Throughout the study, care of the steers was in accordance with guidelines set by the Canadian Council on Animal Care [13].

**Figure 1:**
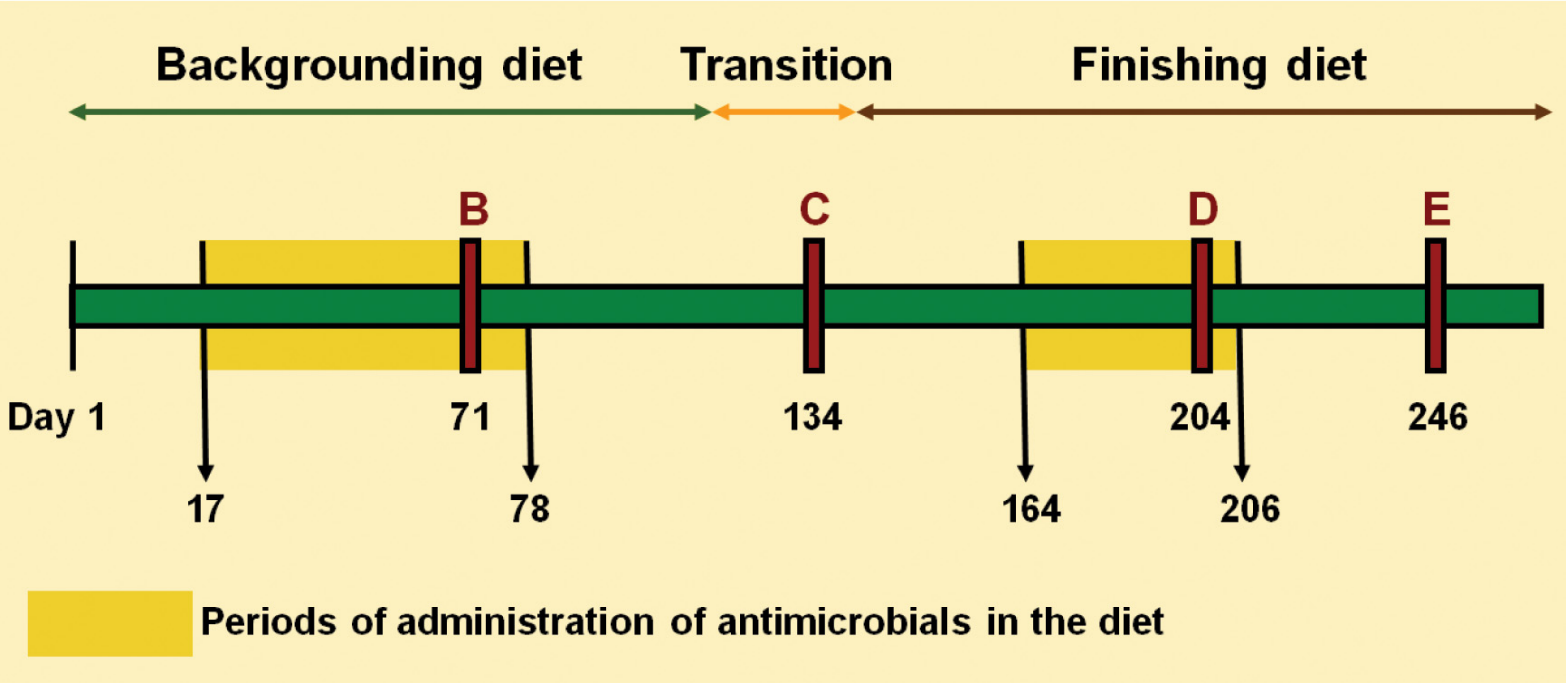
**Feeding and antibiotic administration timeline**. Numbers indicate day of the feeding period and B, C, D, and E represent points where fecal samples were collected from cattle. Silage-based diets were fed for 115 d, followed by 21 d of transition to the grain-based diet, which was then fed until shipment of cattle to market. Shaded areas indicate the periods that antimicrobials were included in the diet.

Diet composition and feeding duration were typical of the feedlot industry in western Canada. A silage-based growing diet containing 70% barley silage, 25% barley grain and 5% vitamin/mineral supplement was fed for 115 days, followed by a step-wise 21-d transition to a grain-based finishing diet (85% barley grain, 10% barley silage and 5% supplement) that was fed to slaughter. For two discrete periods indicated in Figure 1, the antibiotics described below were mixed daily into 5 kg of supplement and spread manually (top-dressed) over the feed for each pen immediately after its delivery into the feed bunk.

### Experimental treatments

The larger trial [12] from which this study was drawn included six experimental treatments (control plus five different dietary antibiotics), each fed to five pens of 10 cattle with the exception of the Aureo S-700G treatment which only had four replicate pens. Limited resources for detailed characterization of *E. coli *isolates dictated that we reduce the number of treatments and sampling days examined. As a result, isolates from monesnin and tylosin treatments were not examined. The present analysis includes isolates from only the control group (CON; no antibiotics added to supplement) and three of the five antibiotic treatment groups: 1) chlortetracycline (T), provided as Aureomycin 100-G (Alpharma Inc., Vineland, NJ, USA) fed at 11 ppm; 2) chlortetracycline + sulfamethazine (TS), provided as Aureo S-700G (Alpharma Inc.) fed at 44 ppm; 3) virginiamycin (V), provided as V-Max (Pfizer Animal Health, New York, NY, USA) fed at 31 ppm. The antimicrobial agents were selected based on the commonality of their use in the Canadian feedlot industry and were fed at the concentrations recommended by the manufacturers. Virginiamycin was included in the study because it is not registered for use in Canada and, as a result, neither calves nor their dams would have had prior exposure to this antibiotic.

### Fecal sampling

Fecal samples were obtained by rectal swab of each steer on 11 occasions [12] throughout the feeding period. This paper presents analysis of isolates collected on 5 of the 11 sampling days. The four samplings (Figure 1) were chosen to represent the five phases in the feeding trial: (i) during their first exposure (while being fed silage-based diet); (ii) during the first period of withdrawal of antibiotics (while being fed silage-based diet); (iii) during the second exposure to antibiotics (while fed grain-based diet); and (iv) following the second withdrawal (while fed grain-based diet). These sample days were designated B, C, D and E, respectively.

### Screening for AMR *E. coli*

On each collection day, fecal swabs were transported to the laboratory in brain heart infusion broth (Becton Dickinson, Sparks, MD, USA) containing 20% glycerol (v/v). Fecal slurry from each steer was plated onto five media (one non-selective and four amended with antibiotics) as described by [12]. Colonies selected from those plates were confirmed as *E. coli *using biochemical tests and fatty acid methyl ester (FAME) profiles [14], and isolates from each steer, sampling day and medium of isolation (when available) were selected for archiving. For the present study, isolates cultured on three media were considered: (i) MacConkey agar with no added antibiotics added (as a control, denoted M^C^); (ii) MacConkey agar amended with 4 μg/ml tetracycline hydrochloride (M^T^); and (iii) MacConkey agar amended with 50 μg/ml ampicillin (M^A^). The concentration of tetracycline was set below [15] standards to ensure isolation of tetracycline-resistant *E. coli*. Ampicillin concentration exceeded the CLSI standard, but was needed to curtail overgrowth that was interfering with isolation of distinct colonies. From the M^C^-, M^T^- and M^A^-selected colonies, a collection of 6354 isolates was established.

The present study aimed to investigate approximately 10% of the available isolates. Archived M^T^- and M^A^-selected isolates from 140 animals, including all 50 steers in the dietary control group (CON), and 30 steers from each of treatment groups T, TS and V, were included for further characterization. Isolates from the treatment groups were chosen by randomly selecting six of the 10 animal ID numbers from each of the 15 antibiotic-treated pens. Then, from the archived collections from each of the five sampling days, isolates from only those six steers were selected for further study. In this manner, a total of 531 *E. coli *isolates were identified for the analyses presented in this paper (Table 1). These comprised 55, 361 and 115 isolates selected initially on M^C^, M^T ^and M^A ^media respectively, of which 94, 99, 155, and 183 were obtained on sampling days B, C, D, and E, respectively.

**Table 1:** Distribution of isolates characterized in this study

**Treatment**^**a**^	**Medium used for selection**^**b**^	Number of animals	**Sampling day**^**c**^				Total
			
			B	C	D	E	
CON	M^C^	5	5	5	5	5	20
	M^T^	50	15	19	47	30	111
	M^A^	50	0	8	1	17	26
T	M^C^	3	3	3	2	3	11
	M^T^	30	12	10	27	25	74
	M^A^	30	2	0	1	10	13
TS	M^C^	3	3	3	3	3	12
	M^T^	30	23	26	29	29	107
	M^A^	30	15	14	7	15	51
V	M^C^	3	3	3	3	3	12
	M^T^	30	11	6	25	27	69
	M^A^	30	2	2	5	16	25
**Total**			**94**	**99**	**155**	**183**	**531**

^a ^Steers were fed no antibiotics (control, CON), or chlortetracycline and sulfamethazine (44 ppm; TS); chlortetracycline (11 ppm; T) or virginiamycin (31 ppm; V) administered in two discrete periods (see Figure 1).

^b ^Isolates were collected by plating fecal slurries onto (i) MacConkey agar (MAC) containing no antibiotics (control, M^C^), or amended with tetracycline hydrochloride (4 μg/mL; M^T^) or with ampicillin (50 μg/mL; M^A^).

^c ^Sampling days occurred during each of the four phases of the feeding trial (see Figure 1).

### Antimicrobial susceptibility testing

Using the agar dilution method according to National Clinical and Laboratory Standards Institute (CLSI) guidelines [16], each isolate was tested for susceptibility to 11 antimicrobials (concentrations, μg/ml): amikacin (AMI; 0.5, 1, 2, 4, 8, 16, 32, 64), ampicillin (AMP; 1, 2, 4, 8, 16, 32), ceftriaxone (AXO; 0.5, 1, 2, 4, 8, 16, 32, 64), cefoxitin (FOX; 0.5, 1, 2, 4, 8, 16, 32), cephalothin (CL; 2, 4, 8, 16, 32), chloramphenicol (CHL; 2, 4, 8, 16, 32), gentamicin (GEN; 0.25, 0.5, 1, 2, 4, 8, 16), nalidixic acid (NAL; 0.5, 1, 2, 4, 8, 16, 32), streptomycin (STR; 32, 64), sulfamethoxazole (SMX; 32, 64, 128, 256, 512), and tetracycline (TE; 1, 2, 4, 8, 16, 32). *Escherichia coli *ATCC 25922, *Pseudomonas aeruginosa *ATCC 27853, *Enterococcus faecalis *ATCC 29212 and *Staphylococcus aureus *ATCC 29213 were included in the panels as controls. Determination of antimicrobial resistance breakpoints for *E. coli *was in accordance with CLSI guidelines [17] except for streptomycin, for which a breakpoint of 64 μg/ml was used according to [18]. These data were used to generate a resistance antibiogram (ABG) for each isolate.

### Pulsed-field gel electrophoresis

Restriction (*Xba*I) digested DNA from each isolate was subjected to PFGE according to the PulseNet USA protocol developed for *E. coli *O157:H7 [19] modified as described previously [18]. PFGE banding patterns were analyzed using BioNumerics software program version 2.5 (Applied-Maths, Ghent, Belgium). DNA fragments on each gel were normalized using the *Salmonella enterica *serovar Braenderup "Universal Marker" as a molecular weight standard. Fingerprints were clustered into groups using Dice coefficient and evaluated by the unweighted-pair group method. All isolates in a single cluster (≥ 90% homology) were considered to be from a similar source and genetically related, as previously described [20] and Tenover et al., 1995 F.C. Tenover, R.D. Arbeit, R.V. Goering, P.A. Mickelsen, B.E. Murray, D.H. Persing and B. Swaminathan, Interpreting chromosomal DNA restriction patterns produced by pulsed-field gel electrophoresis: criteria for bacterial strain typing, *Journal of Clinical Microbiology ***33 **(1995), pp. 2233-2239. View Record in Scopus | Cited By in Scopus (4225)[21] and were assigned an arbitrary classification letter to enable temporal and phenotypic trends to be evaluated.

### Multiplex PCR for tetracycline- and ampicillin-resistant isolates

From each cluster in which the PFGE patterns and ABG were identical among member isolates, a single isolate was randomly selected for characterization of tetracycline- and β-lactamase resistance determinants. Isolates not grouped in a cluster, and those that grouped into clusters containing isolates with differing ABG patterns, were also subjected to molecular characterization of resistance determinants. Resistance determinates were chosen based on upon genes that have been commly reported in *E. coli *[22] including genes *tet*(A), *tet*(B), *tet*(C) and others that are not commonly detected among *E. coli *including [23,24]*tet*(D), *tet*(E), *tet*(G), *tet*(K), *tet*(L), *tet*(M), *tet*(O), *tet*(S), *tet*(Q), *tet*(X), and *tet*A(P); and the ampicillin-resistant *E. coli *were screened for the β-lactamase genes *oxa1*-like, *pse-1*, and *tem1*-like. The tetracycline genes were grouped as described by [25] into Group I: *tet*(B), *tet*(C), *tet*(D); Group II: *tet*(A), *tet*(E), *tet*(G); Group III: *tet*(K), *tet*(L), *tet*(M), *tet*(O), *tet*(S); and Group IV: *tet A*(P), *tet*(Q), *tet*(X). Primer pairs were selected from previously published sources [25-29] and the expected amplicon sizes are listed in Table 2.

**Table 2:** Primers used in assay of isolates for resistance determinants

Gene	**PCR primer sequence 5'-3' **^**a**^	Amplicon size (bp)	Genbank accession no.	Control plasmid/gDNA	Source of plasmid and reference
*tet*(A)	GCT ACA TCC TGC TTG CCT TC	210	X61367	pSL18	[25]
	CAT AGA TCG CCG TGA AGA GG				
*tet*(B)	TTG GTT AGG GGC AAG TTT TG	659	J01830	pRT11	[25]
	GTA ATG GGC CAA TAA CAC CG				
*tet*(C)	CTT GAG AGC CTT CAA CCC AG	418	J01749	pBR322	[25]
	ATG GTC GTC ATC TAC CTG CC				
*tet*(D)	AAA CCA TTA CGG CAT TCT GC	787	L06798	pSL106	[25]
	GAC CGG ATA CAC CAT CCA TC				
*tet*(E)	AAA CCA CAT CCT CCA TAC GC	278	L06940	pSL1504	[25]
	AAA TAG GCC ACA ACC GTC AG				
*tet*(G)	GCT CGG TGG TAT CTC TGC TC	468	S52437	pJA8122	[25]
	AGC AAC AGA ATC GGG AAC AC				
*tet*(K)	TCG ATA GGA ACA GCA GTA	169	S67449	PAT102	[25]
	CAG CAG ATC CTA CTC CTT				
*tet*(L)	TCG TTA GCG TGC TGT CAT TC	267	U17153	pVB.A15	[55]
	GTA TCC CAC CAA TGT AGC CG				
*tet*(M)	GTG GAC AAA GGT ACA ACG AG	406	X90939	pJ13	[25]
	CGG TAA AGT TCG TCA CAC AC				
*tet*(O)	AAC TTA GGC ATT CTG GCT CAC	515	Y07780	pUOA1	Taylor^b^
	TCC CAC TGT TCC ATA TCG TCA				
*tet*(S)	CAT AGA CAA GCC GTT GAC C	667	C92946	pAT451	Mulvey
	ATG TTT TTG GAA CGC CAG AG				
*tet*A(P)	CTT GGA TTG CGG AAG AAG AG	676	L20800	pJIR39	Monash University^c^
	ATA TGC CCA TTT AAC CAC GC				
*tet*(Q)	TTA TAC TTC CTC CGG CAT CG	904	X58717	pNFD13-2	Salyers^d^
	ATC GGT TCG AGA ATG TCC AC				
*tet*(X)	CAA TAA TTG GTG GTG GAC CC	468	M37699	pBS5	[56]
	TTC TTA CCT TGG ACA TCC CG				
*pse-*1	CGC TTC CCG TTA ACA AGT AC	419	M69058	SU01	[28]
	CTG GTT CAT TTC AGA TAG CG			gDNA	
*oxa1*-like	AGC AGC GCC AGT GCA TCA	708	AJ009819	SU05	[26]
	ATT CGA CCC CAA GTT TCC			gDNA	
*tem1*-like	TTG GGT GCA CGA GTG GGT	503	AF126482.1	SU07	[26]
	TAA TTG TTG CCG GGA AGC			gDNA	

^a ^Primers selected from previously published source [26,26].

^b ^Provided by Dr.Taylor (University of Alberta, Edmonton, AB, Canada).

^c ^Provided by the Monash University (Victoria, Australia).

^d ^Provided by Dr. Salyers (University of Illinois, Urbana, USA).

For PCR amplifications, bacterial cells from a single colony were collected using a sterile toothpick and resuspended in 25 μl of sterile deionized water. Amplifications were carried out in a Dyad PCR system (Bio-Rad Laboratories, Inc., Mississauga, ON, Canada) as described by [18]. PCR mixture (total 25 μl) included 1 μl of DNA template, 1 × PCR buffer (Invitrogen), 2.5 U Platinum *Taq *polymerase (Invitrogen) 300 μM of dNTP (Invitrogen) and sterile deionized water. Primers and MgCl_2 _concentrations for the tetracycline group were optimized as described by [25]; for the ampicillin *g*roup, *pse-1 *(1.0 μM), *oxa1*-like (1.0 μM), *tem1*-like (1.0 μM), and 3.0 mM MgCl_2 _were used. For the tetracycline group, PCR conditions were: 5 min denaturing at 94°C; 28 cycles of 94°C for 1 min, 59.5°C for 1 min and 72°C for 1.5 min; final extension 5 min at 72°C. For the ampicillin group, denaturing was 5 min at 94°C, then 25 cycles of 94°C for 30 sec, 60°C for 30 sec and 72°C for 40 sec, and final extension 5 min at 72°C. PCR products were analyzed by gel electrophoresis on a 1.5% (w/v) agarose gel in 1× TAE buffer. DNA bands were stained with ethidium bromide and visualized by UV transillumination. Reference *E. coli *cultures and *Salmonella typhimurium *control plasmids and genomic DNA (gDNA) possessing tetracycline- and ampicillin-resistance genes (Table 2) were included, as well as a 100-bp DNA ladder (Invitrogen) for assessing size of PCR products.

### Statistical analysis

The GENMOD procedure (SAS Institute Inc. 2008) was used to perform a log-linear analysis separately for each medium to evaluate differences among recovered isolates for antimicrobial resistance phenotypes, treatments and their interaction. *P *values ≤ 0.05 were interpreted as indicative of a significant difference.

PFGE patterns were either classified as unique or grouped into clusters based on ≥ 90% homology using Dice similarity coefficients using unweighted pair group methods with arithmetic average algorithms built into Bionumerics. The position tolerance and optimization were set at 1% and 0.5% respectively.

## Results

### Antimicrobial susceptibility

Resistance to AMI, FOX, AXO, GEN, or NAL was not observed in any of the 531 *E. coli *isolates examined (isolated on M^C^, M^T ^or M^A^).

#### Populations selected on M^c ^plates

Forty-five of 55 isolates (81.8%) from non-selective medium M^C ^were susceptible to all antimicrobials tested. Phenotypes observed in the M^C ^isolates expressing AMR included resistance to SMX (7/10 isolates), STR (5/10), CHL (2/80), TE (2/10) and CL (1/10). Six of the 10 isolates obtained exhibited multi-drug resistance.

#### Populations selected on M^T ^plates

Resistance to TE at the breakpoint level was nearly ubiquitous (>98.8%) among the isolates from the M^T ^plates (Table 3). Isolates from M^T ^plates exhibiting AMP, STR, SMX and TE were recovered from animals across all three treatments. A treatment × phenotype interaction (p = 0.003) was observed with an increased number of isolates (p = 0.014) exhibiting resistance to SMX in TS group (55.1%) as compared to other groups (Table 3). Resistance to STR was higher (p = 0.018) among CON (52.3%) and V (50.7%) groups as compared to T (35.1%) and TS (32.7%) treatments (Table 3). Resistance of M^T ^isolates to AMP was highest (p = 0.017) in isolates recovered from TS (18.7%) and was less common among isolates from groups V (13.0%), CON (6.3%) and T (2.7%).

**Table 3:** Total number (n) and percentage of phenotype observed within isolates recovered from MacConkey agar amended with 4 μg/ml tetracycline hydrochloride after diet administration of control and three antimicrobial treatments.

	**Treatment**^**†**^			
**Phenotype**	**CON % (*n*)**	**T % (*n*)**	**TS % (*n*)**	**V % (*n*)**
AMP	6.3^b ^(7)	2.7^c ^(2)	18.7^a ^(20)	13.0^b ^(9)
STR	52.3^a ^(58)	35.1^b,c ^(26)	32.7^b ^(35)	50.7^a ^(35)
SMX	42.3^c ^(47)	47.3^b,c ^(35)	55.1^a ^(59)	42.0^b ^(29)
TE	99.1^ba ^(110)	100^a ^(74)	100^a ^(107)	98.6^b ^(68)
**Total (*n*)**	111	74	107	69

^†^CON; no antibiotics added to supplement, T: chlortetracycline provided as Aureomycin 100-G fed at 11 ppm, TS: chlortetracycline + sulfamethazine, provided as Aureo S-700G (Alpharma Inc.) fed at 44 ppm and V: virginiamycin provided as V-Maxed at 31 ppm.

#### Population selected on M^A ^plates

As expected, given that the concentration of ampicillin in the selection medium was above the breakpoint level, resistance to AMP was confirmed in all of M^A ^isolates (Table 4). Isolates exhibiting resistance to TE, CL and STR were obtained from cattle fed all diets. Resistance to TE phenotype was higher (p <0.001) in M^A ^isolates from TS (94.1%) as compared to T (76.9%) and V (56.0%) and CON (38.5%) steers (Table 4). In the M^A ^isolates from CON, resistance to CL was most common, and its prevalence (61.5%) was notably higher (p = 0.007) than was observed in the T (15.4%), TS (5.9%) or V (4.0%) isolates (Table 4).

**Table 4:** Total number (n) and percentage of phenotype observed within isolates recovered from MacConkey agar amended with 50 μg/ml ampicillin after diet administration of control and three antimicrobial treatments.

	**Treatment**^**†**^			
**Phenotype**	**CON % (*n*)**	**T % (*n*)**	**TS % (*n*)**	**V % (*n*)**
AMP	100 (26)	100 (13)	100 (51)	100 (25)
CL	61.5^a ^(16)	15.4^b ^(2)	5.9^b ^(3)	4.0^b ^(1)
STR	38.5 (10)	23.1 (3)	13.7 (7)	40.0 (10)
TE	38.5^c ^(10)	76.9^b ^(10)	94.1^a ^(48)	56^c ^(14)
**Total (*n*)**	26	13	51	25

^† ^CON; no antibiotics added to supplement, T: chlortetracycline provided as Aureomycin 100-G fed at 11 ppm, TS: chlortetracycline + sulfamethazine, provided as Aureo S-700G (Alpharma Inc.) fed at 44 ppm and V: virginiamycin provided as V-Maxed at 31 ppm.

### Antibiogram patterns

**Figure 2:**
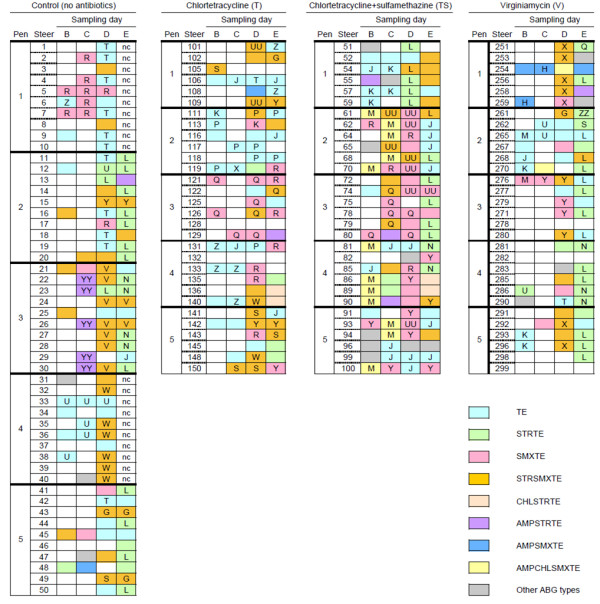
**Antibiogram and PFGE types of fecal *E. coli *isolated from feedlot cattle using MacConkey agar amended with 4 μg/ml chlortetracycline (M^T^), as distributed by dietary treatment, sampling day and animal of origin**. Sampling days (B to E) are depicted in Figure 1. Each box represents a single isolate from a particular steer on a given sampling day. The first eight colors represent the most commonly observed antibiogram patterns with grey indicating an infrequently observed antibiogram. Unfilled boxes indicate no isolate obtained on M^T^. Common letters indicate isolates with >90% genetic homology. Shaded boxes without a letter indicate isolates with <90% genetic homology with antibiogram data. Dietary treatments were as follows: Control: no antibiotics; Chlortetracycline (11 ppm; denoted T); Chlortetracycline + sulfamethazine (44 ppm; denoted TS); and Virginiamycin (31 ppm; V). nc: isolates not characterized.

**Figure 3:**
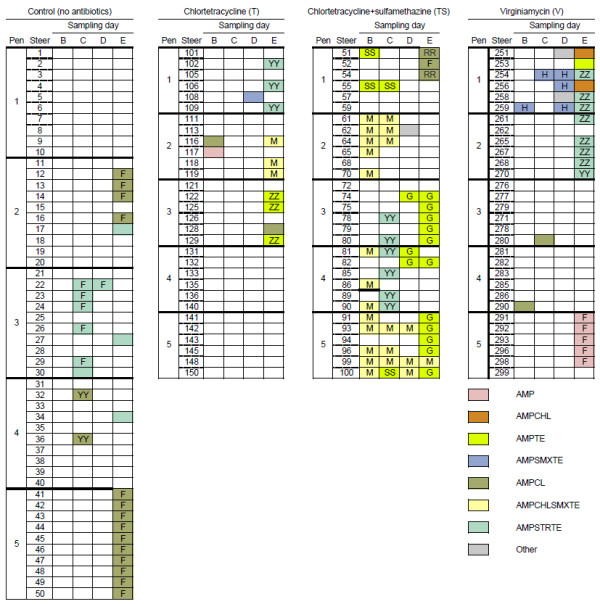
**Antibiogram and PFGE types of fecal *E. coli *isolated from feedlot cattle using MacConkey agar amended with 50 μg/ml ampicillin (M^A^), as distributed by dietary treatment, sampling day and animal of origin**. Sampling days (B to E) are depicted in Figure 1. Each box represents a single isolate from a particular steer on a given sampling day. The first eight colors represent the most commonly observed antibiogram patterns and grey indicates an infrequently observed antibiogram. Unfilled boxes indicate no isolate was obtained on M^A^. Common letters indicate isolates with >90% genetic homology. Shaded boxes without a letter indicate isolates with <90% genetic homology with antibiogram data. Dietary treatments were as follows: Control: no antibiotics; Chlortetracycline (11 ppm; denoted T); Chlortetracycline + sulfamethazine (44 ppm; denoted TS); and Virginiamycin (31 ppm; V).

Irrespective of the CON or antibiotic treatment administered, the majority of isolates, particularly those from M^A ^medium, were resistant to multiple antibiotics. Among the M^T ^isolates, multi-resistance whereby a single isolate displayed resistance to more than one antibiotic, was found in 69.4%, 56.8%, 76.6% and 73.9% of CON, T, TS and V isolates, respectively (Figure 2). By comparison, in the M^A ^isolates, multi-resistance was observed in 100, 92.3, 100, and 80.0% of isolates from CON, T, TS and V steers, respectively (Figure 3).

#### Population selected on M^T^

The ABG patterns of M^T ^isolates from steers in the CON and V treatments were similar (Figure 2). In both treatments, M^T ^isolates with the STRSMXTE pattern were obtained primarily on sampling day D (in 22 CON isolates, and 12 from group V). In a similar fashion, the STRTE pattern was detected in M^T ^isolates primarily on sampling day E (*n *= 18 and *n *= 17 in CON and V, respectively). The STRTE ABG pattern was not found in the CON isolates from pens 1 or 4, but STRTE isolates were recovered from all 5 pens in group V. From the V steers, 10 of 18 M^T ^isolates from pen 2 exhibited the TE pattern. Four M^T ^isolates with pattern AMPSMXTE were obtained from V steers in pen 1, whereas among isolates from CON steers, this pattern was identified only once (steer 48, day C). Antibiogram AMPSTRTE was identified in isolates from 5 CON steers in pen 3 on day C. The SMXTE phenotype was observed more commonly in CON isolates than in those from group V, notably in those collected in pen 1, where 8 of 18 isolates obtained exhibited SMXTE.

The TE phenotype accounted for 17 of 22 isolates collected from steers fed T during the growing phase (silage-based diet; days B and C), compared with only 15 of 52 isolates collected during grain feeding (days D and E). During that period, observation of SMXTE (12/52) and STRSMXTE (17/52) in M^T ^isolates from group T was more frequent than it had been earlier (3 SMXTE and 2 STRSMXTE isolates from group T on days B and C). The SMXTE pattern was recovered mainly from pen 3, whereas M^T ^isolates with pattern STRSMXTE were more widely distributed across pens, particularly on day D.

The ABG patterns of M^T ^isolates from TS steers early in the feeding period (sampling days B and C) differed from isolates collected later (Figure 2). For example, the AMPCHLSMXTE pattern was observed on days B (*n *= 7) and C (*n *= 5), but not on days D or E. In contrast, few isolates with the SMXTE pattern were obtained from TS steers on sampling days B (*n *= 3) and C (*n *= 4). By sampling day D, however, this ABG was predominant among TS isolates (*n *= 17) in all pens except pen 1. Also in the TS group, M^T ^isolates with ABG pattern STRTE were obtained more frequently on later (grain-based diet) sampling days (D; *n *= 4 (all in pen 1) and E; *n *= 7) as compared to isolates collected earlier, during feeding of silage-based diet (0 and 2 isolates from days B and C, respectively, exhibited STRTE). Isolates exhibiting the STRSMXTE antibiogram were widely distributed among M^T ^isolates, as were those with the TE phenotype.

#### Population selected on M^A^

Two ABG types (AMPCL and AMPSTRTE) were observed in M^A ^isolates collected from CON steers, and these tended to cluster with sampling day and pen of origin (Figure 2). On day C, AMPSTRTE was predominant, observed in 6 of 8 isolates expressing AMR, all in pen 3. On this sampling day, the two AMR isolates from pen 4 had AMPCL phenotype. On day E, AMPSTRTE isolates were also recovered from adjacent pens 2 and 4, but AMPCL pattern was predominant, both in pen 2 (4 of 5 AMR isolates) and particularly in pen 5 (10 of 10).

From steers in group T, M^A ^*E. coli *isolates were relatively uncommon, with the majority (10/13) occurring only on day E (Figure 2). In this group, ABG patterns were distinctly associated with specific pens. Phenotypes AMPSTRTE, AMPCHLSMXTE, and AMPTE (each *n *= 3) were exclusive to pens 1, 2 and 3, respectively.

More M^A ^isolates were associated with steers in group TS than with CON, T or V (Table 1; Figure 2), and the TS isolates were more routinely recovered across all sampling days, whereas in the other groups, isolation was more frequent later in the feeding period (days D, E) compared with the growing phase (days B, C). As with the CON isolates, sampling time and pen of origin influenced the likelihood with which M^A ^isolates with a specific ABG were observed. The AMPCHLSMXTE phenotype was most common (23 of 51 isolates) in the TS group. It was observed primarily on the earlier sampling days (19/23 on days B and C), and exclusively in pens 2, 4 and 5 on day B. Late in the feeding period (grain-based diet; day E), phenotype AMPTE was prevalent (in 11 of 15 isolates from that day, clustered mainly in pens 3 and 5).

The ABG patterns characterized from the M^A ^isolates from V steers was also dependent on the sampling time as well as the pen (Figure 3). For example, with the exception of steer 117 in treatment T, sampling B, M^A ^isolates with ABG pattern AMP were obtained exclusively during sampling E from five V steers in pen 5 (Figure 3). Similarly, M^A ^isolates with ABG pattern AMPCHL were isolated exclusively at sampling E from two V steers housed in pen 1, and 8 isolates with ABG pattern AMPSTRTE were isolated at sampling E from steers in adjacent pens 1 and 2. Finally from the V group, M^A ^isolates with ABG pattern AMPSMXTE were obtained only from pen 1 during sampling B, C and D.

### PFGE types

A large number of PFGE genotypes were detected from throughout the feedlot, in all treatments. Many of these genotypes were isolated only transiently during the feeding period. The M^T^-selected isolates in groups CON, T, TS and V presented 46, 37 35 and 34 PFGE genotypes. Among the M^A ^isolates from CON, T, TS, V samples, 8, 7, 7, and 11 PFGE genotypes, respectively, were identified.

#### Population selected on M^T^

Unlike the M^A ^isolates, many of the M^T ^isolates with the same ABG exhibited two or more different PFGE profiles (Figure 2). For example, from CON, the TE isolates exhibited 19 different PFGE banding patterns, with two predominant patterns that were associated with pens 1 and 4. Isolates with ABG patterns STRSMXTE and STRTE obtained from CON steers also frequently exhibited different PFGE types. Of note, although the PFGE genotypes of STRSMXTE isolates in pens 3 and 4 clearly differed between pens, within pen, the majority of these isolates (9/11 in pen 3 and 6/7 in pen 4) were clones. All of the AMPSTRTE isolates from CON steers, with the exception of one isolate from pen 2, were associated with pen 3 and possessed indistinguishable PFGE patterns. Clonal isolates with the STRTE phenotype were also obtained from CON steers in pens 2, 3 and 5 during later samplings, but STRTE *E. coli *exhibiting different PFGE profiles were also present in pen 2 and pen 3.

In group T, M^T ^isolates with the TE phenotype exhibited 16 different PFGE profiles (Figure 2), though within a pen, these isolates often exhibited the same PFGE profile (e.g., 7 of 12 TE isolates in pen 2 were indistinguishable, as were 4 of 7 in pen 4). The isolates with SMXTE phenotype also clustered by pen: 6 of 8 in pen 3 were indistinguishable, as were all three SMXTE isolates from pen 4. Throughout the feeding period, the TE isolates from diet group T tended to exhibit three predominant PFGE types. As the frequency of isolation of STRSMXTE isolates increased in the finishing feeding period, so too did the diversity of their PFGE types. The two isolates from days B and C (growing period) were indistinguishable, whereas 10 PFGE patterns were identified among the 17 STRSMXTE isolates from days D and E (finishing period).

In the TS group, the SMXTE ABG occurred frequently in all pens except pen 1 and was represented by 10 different PFGE profiles across pens (Figure 2) and all 10 were recovered on day D. Overall, the SMXTE isolates exhibited three main PFGE profiles. Similarly, the TS isolates with STRSMXTE phenotype were associated with 11 PFGE types, with diversity evident particularly in pen 1. A PFGE profile (J) that was also identified in TE isolates from diet group T, was the predominant PFGE type among the TE isolates from diet group TS, identified in 14 of the 25 isolates with that phenotype. These indistinguishable isolates were associated primarily with pens 2 and 5, and were not recovered from pen 3. The STRTE isolates from pens 1 and 3 (and the sole STRTE isolate in pen 2) were indistinguishable, whereas this phenotype was not observed in pen 5, and the four STRTE isolates in pen 4 exhibited different PFGE profiles. All 12 M^T ^isolates with AMPCHLSMXTE phenotype, clustered in pens 2, 4 and 5, exhibited indistinguishable PFGE profiles.

#### Population selected on M^A^

Among the M^A ^isolates, most that exhibited a given ABG pattern also presented indistinguishable PFGE profiles (Figure 3). In the CON group, 14 of the 16 AMPCL isolates, collected from pens 2 and 5, had indistinguishable PFGE profiles. Similarly, 6 of the 10 AMPSTRTE M^A ^isolates from CON cattle were clones and associated only with pen 3.

As with the AMPCHLSMXTE isolates from M^T^, the M^A ^isolates displaying this phenotype were all found to possess indistinguishable PFGE profiles, and were obtained primarily from steers on the TS treatment (Figure 3). Steer 99 (pen 5) was the only animal from which the same AMR clone was recovered on all four sampling days. The AMPTE isolates from group TS exhibited two distinct PFGE profiles - a predominant type recovered in pens 3, 4 and 5, and the second type from pen 1 with the exception of one isolate in pen 5. The phenotype AMPSTRTE was associated with only a single PFGE profile, and only in pens 3 and 4 on day C.

The PFGE profiles of AMPSTRTE and AMPCHLSMXTE isolates recovered from group T steers on day E were indistinguishable from those determined in the TS group, but the AMPTE isolates (3 clones in pen 3) exhibited a distinct PFGE to that of the AMPTE isolates from TS.

Similarly, associations of single PFGE profiles with specific ABG patterns were found among most of the M^A ^isolates from diet group V, and mainly on day E. All of the AMP isolates obtained from steers in pen 5 were clones, as were 4 of the 5 AMPSTRTE isolates from pen 2, and 3 of 3 in pen 1. All five AMPSMXTE isolates from pen 1 (across three sampling days) exhibited indistinguishable PFGE profiles.

### Multiplex PCR

Tetracycline genes only from Group I [*tet *(B), *tet *(C), *tet *(D)] and Group II [*tet *(A), *tet *(E), *tet *(G)] were identified, with no genes from Group III [*tet *(K), *tet *(L), *tet *(M), *tet *(O), *tet *(S)] or Group IV [*tet A *(P), *tet *(Q), *tet *(X)] being detected in any of the isolates examined. The *tet*(B) gene was the most commonly observed of the tetracycline resistance determinants, present in 58.2%, 53.5%, 40.8% and 50.6% of M^T ^isolates from CON, T, TS, and V steers, respectively. The *tet*(A) determinant was detected in 22.5%, 51.4% and 26.0% of the isolates from T, TS and V, respectively, but was present in only 12.2% of the isolates from CON. Determinant *tet*(C) was also present at low frequencies, detected in 7.1, 12.7, 2.1 and 13.0% of M^T ^isolates from groups CON, T, TS and V, respectively. A small proportion of the isolates examined, 20.4, 5.6 and 2.6% from CON, T and V, respectively, did not possess any of the tetracycline determinants screened for. Few isolates possessed multiple tetracycline resistance determinants. The *tet*(A) and *tet*(B) genes were present together in only 0.7% of the isolates from the TS group, and 0.8% of the isolates from CON. Combinations of *tet*(B) and *tet*(C) were detected in 2.0, 5.6, 4.9 and 6.5% of the M^T ^isolates from CON, T, TS and V. The *tet*(A) and *tet *(C) were detected in combination in only 1.3% of M^T ^isolates from steers in group V.

Ampicillin-resistant isolates from all treatment groups were subjected to multiplex PCR to ascertain the presence of *bla*_PSE-1_, *bla*_OXA1 _and *bla*_TEM-1 _determinants. The *bla*_TEM-1 _determinant was present in 50.0, 66.7, 80.3 and 100% of M^A ^isolates from the CON, T, TS and V groups, respectively. The other ampicillin resistance determinants that were screened were not detected in 54.4% of the other M^A ^isolates.

## Discussion

Chlortetracycline alone and combined administration of chlortetracycline and sulfamethazine were selected as experimental treatments on the basis of their routine use in the Canadian feedlot industry. These antimicrobials are used to improve feed efficiency and prevent foot rot, liver abscesses and respiratory disease. Virginiamycin was included in the study as an antibiotic to which neither the steers nor their dams would have had prior exposure, given that it is not registered for use in cattle in Canada.

Resistance to amikacin, ceftriaxone (64 μg/ml), cefoxitin or nalidixic acid was not detected in any of the 531 *E. coli *isolates examined. Other researchers of *E. coli *from Canadian beef cattle have also reported the absence of resistance to these antibiotics [30] or, when resistance to nalidixic acid was found, it occurred in fewer than 2% of isolates studied [31]. In the present study, the absence of resistance to these antibiotics in gut flora may be related to sole-source acquisition of the calves, and to the complete absence of antibiotic use prior to their arrival at the feedlot. Furthermore, our research feedlot had been constructed just prior to commencement of this experiment, thus there was no history of prior administration of subtherapeutic antibiotics at this site. Our results and those of others [30,31] contrast with those of Hoyle et al. [32], who reported that all calves from a Scottish beef farm were found to shed nalidixic acid-resistant *E. coli *at least once during a 21-wk study.

Comparisons of AMR *E. coli *from steers in CON vs. T, TS and V groups suggests that subtherapeutic administration of these antimicrobials had only a limited impact on the nature of antimicrobial resistance in *E. coli *resident in these cattle. The resistances observed most commonly among these *E. coli *isolates were to tetracycline, sulfamethoxazole, ampicillin, chloramphenicol and streptomycin, which is consistent with the findings of other Canadian beef researchers [30,31,33].

In general, the antibiogram type and temporal point of isolation were more similar between isolates from CON and V groups than from those in T or TS. Virginiamycin, a streptogramin, that primarily targets Gram-positive bacteria [34], and appears to have had minimal influence on the nature of AMR in the non-target *E. coli *isolates obtained in this study. Similarly, dietary inclusion of monensin, another antibiotic that targets Gram-positive bacteria, also did not alter the nature of AMR *E. coli *isolated from beef cattle [35]. These results suggest that antimicrobial suppression of Gram-positive bacteria does not give rise to unoccupied microbial niches that are filled via AMR *E. coli*.

Despite the fact that the *E. coli *characterized in this study were recovered from selective media, the fact that antibiotic resistance, particularly to tetracycline, streptomycin, sulfamethoxazole and ampicllin, was common in *E. coli *from cattle that were not administered tetracycline suggests that naturally occurring resistance determinants circulate in bovine gut microbial populations for reasons other than selection as a result of antimicrobial agents being included in the diet. Hoyle et al. [36] characterized bovine fecal *E. coli *from an organic farm and found that even with the restricted use of antimicrobials, ampicillin-resistant *E. coli *were readily isolated. In that study, age of the cattle and likely the diet they were provided, as opposed to subtherapeutic administration of antibiotics appeared to be an important factor for the acquisition and development of antibiotic-resistant commensal microflora. A higher prevalence of AMR *E. coli *in feces from younger than older animals within the same farm has been previously reported [37,38]. A comprehensive longitudinal study of four feedlots in which antibiotics were only used therapeutically also found no difference in the nature of AMR among isolates collected from home pens compared with those from hospital pens in which antibiotics were administered [39]. Our work as well as that of others has also observed that the presence and dissemination of AMR in *E. coli *during the feeding period may be a response to the diet rather than antimicrobial administration [12,18,40]. In the present study, short-term withdrawal of antibiotics appeared to have minimal impact on AMR in *E. coli*, given that AMR isolates were collected routinely on days C and E. Perhaps this is not surprising when one considers that even long term withdrawal of antimicrobials has in some cases had minimal impact on the nature of antimicrobial resistance [41]. In the case of genetic determinants for tetracycline resistance, it has been proposed that these elements have established a steady state in *E. coli *populations, and that their presence is not necessarily related to antimicrobial usage [22].

Perhaps the most obvious impact of antimicrobial administration on the phenotype and genotype of *E. coli *was observed for isolates obtained from TS fed cattle, a response that may reflect the fact that two antimicrobials were administered to these animals. The M^T ^isolates from the TS group exhibited a higher frequency of SMX resistance and as both sulfamethazine and sulfamethoxazole (SMX) are sulfonamides, this may reflect selection for strains resistant to SMX. Sharma et al. [20] recently reported similarities in the numbers of ampicillin-resistant and tetracycline-resistant isolates, as well as the types of resistance phenotypes observed, in *E. coli *collected from cattle fed chlortetracycline (44 ppm) alone or in combination with sulfamethazine at the same concentration. These results suggest that the administration of chlortetracycline, even in the absence of sulfamethazine, can lead to the emergence of resistance to SMX, as well as other antibiotics, including AMP and CHL.

*E. coli *exhibiting STRSMXTE and SMXTE resistance phenotypes have been frequently isolated from cattle [42]. Enne et al. [43] documented that the prevalence of sulfonamide resistance among *E. coli *remained constant even with a 97% reduction in the clinical use of sulfonamides in the UK. Further work showed that a plasmid carrying the resistance determinants *sul*2, *str*A and *str*B enhanced host fitness even in the absence of antibiotic selective pressure [44]. Linkages between CHL and TE phenotypes, sulphonamide resistance, and other resistance determinants have been described in plasmid profiling of human clinical isolates in Australia [45], but at this point it remains to be determined if similar linkages are responsible for the linked dissemination of these resistances in feedlot cattle. It is also possible that genes that confer fitness to environmental challenges (e.g., acid tolerance, nutrient limitations, metal concentration) other than those imposed by antibiotics are harboured on these plasmids and promote the acquisition of resistance determinants [46].

Detection of specific AMR *E. coli *frequently appeared to be transient over the duration of this study. Only in one steer (ID 99; group TS) was the same AMPCHLSMXTE *E. coli *clone obtained on all 4 sampling days. Others have also reported that the majority of *E. coli *O157:H7 subtypes occur intermittently within cattle and that few isolates persist for extended periods of time [47]. Although isolates occurred transiently, there were instances where a particular isolate clearly occurred more frequently during specific phases of the feeding period. For example, *E. coli *isolates exhibiting STRTE phenotype were recovered almost exclusively on days D and E, particularly from CON, TS and V steers, and the majority of isolates were clones. This suggests that this particular isolate disseminated readily among pen mates within the feedlot or that this particular clone may have possessed fitness attributes that promoted its prevalence at these points during the feeding period.

In some instances, the occurrence of clones was clearly pen-associated. Some M^T^-isolated *E. coli *clones with specific PFGE profiles occurred exclusively or nearly exclusively within a single pen (e.g., STRSMXTE with PFGE type X in pen V-1). This same phenomenon was also observed for *E. coli *isolates with ampicillin resistance, i.e., cultured on M^A ^(e.g., AMP with PFGE type F, pen V-5). The association of isolates with specific pens was not solely related to the administration of antibiotics, given that some pen associations were evident in the CON group as well (AMPSTRTE with PFGE type YY in pen CON-3; STRSMXTE with PFGE type W in pen CON-4). These findings suggest that the degree of transference of AMR *E. coli *in the feedlot depends on the subtype in question. A previous study in or laboratory used genotyping to document movement of *E. coli *strains from animal to animal within the feedlot environment [20]. Others have reported that housing location can influence the nature of antimicrobial resistance in fecal coliforms from swine [48], but in that study, the pigs were housed in different barns as opposed to different pens within a common building. We determined previously that a rifampin-resistant strain of *E. coli *was transferred infrequently among feedlot cattle housed in adjacent pens even when it was inoculated (10^10 ^CFU) into Trojan steers [49]. In the present study, there was possible evidence of transmission of ampicllin-resistant *E. coli *among adjacent pens as identical AMPTE subtypes were recovered from TS steers in pens 3, 4, and 5 sampled on day E. Similarly, identical AMPSTRTE subtypes were obtained from V steers in adjacent pens 1 and 2 during this same sampling period. Our results suggest that the pen boundaries act as a significant impediment to the widespread dissemination of some AMR *E. coli *subtypes within the feedlot. At this point it is not known if a similar phenomenon would be observed in all feedlots as our feedlot only represented a single ecological unit.

Resource constraints limited our characterizations to only single isolate from each selective plate from each steer during later samplings. It further restricted our ability to study isolates from all steers on all treatments It is possible that this approach may not have given a complete picture of the genetic diversity of tetracycline- and ampicillin-resistant *E. coli *present in feedlot steers. Ensuring representative sampling is always a challenge considering the voluminous nature of digesta within the bovine intestinal tract and the number of cattle that are typically housed within a feedlot. Others have reported that examining single vs multiple isolates did not compromise interpretation of the temporal trends or the nature of diversity of *E. coli *within cohorts [50,51]. In early samples, where we did select two isolates, PFGE frequently identified both isolates as clones. That finding is perhaps not surprising, given the frequency with which we isolated clones from individual pen mates. This pattern may have been amplified by the use of selective plates for establishing the isolate collections, a practice that obviously selects for less diverse subpopulations. In the present study, the degree of diversity was clearly related to the nature of the resistant phenotype. Some phenotypes such as TE, SMXTE and STRSMXTE exhibited a high degree of diversity whereas others, such as AMPCHLSMXTE were solely of a clonal nature suggesting the resistance genes may be chromosomally encoded while others may be plasmid mediated both of which could contribute to the varying degrees of diversity among isolates examined.

Screening for resistance determinants showed that the majority of tetracycline-resistant isolates harboured the *tet*(B) efflux gene, followed less frequently by *tet*(A) and *tet*(C). These findings are consistent with those of Walk et al. [22] who reported that 64.8%, 28.1 and 4.6 of tetracycline-resistant isolates from conventional and organic dairies possessed *tet*(B), *tet*(A) and *tet*(C) determinants. The prevalence of *tet *efflux genes in *E. coli *is likely related to their occurrence on mobile conjugative plasmids and transposons, although *tet*(B) has recently been reported also to integrate into chromosomal DNA [52]. *Tet*(B) has been reported in a variety of other Gram-negative bacteria, including *Enterobacter*, *Proteus*, *Salmonella*, *Actinobacillus*, *Haemophilus, Morazella *and *Treponema *spp. This distribution is thought to reflect frequent gene transfer [52]. In the present study, isolates from M^T ^were screened for other efflux, ribosomal protection, and tetracycline catabolism determinants that included *tet*(K), *tet*(L), *tet*(M), *tet*(O), *tet*(S), *tet*A(P), *tet*(Q), and *tet*(X). This group of *tet *genes are normally present on mobile conjugative plasmids or chromosomally located in Gram-positive bacteria [23], but there has been reports of their transfer to phylogenetically distant bacteria, as *tet*(K) and *tet*(L) have been reported in Gram-negative bacteria [24]. Our screening failed to detect these genes, and to our knowledge, there have been no reports of these determinants occurring in *E. coli*.

During screening of the ampicillin-resistant isolates for three β-lactamase genes the *bla*_TEM1 _determinant was detected in 50 to 100% of isolates from the four treatment groups. Amplicons for *bla*_OXA1 _or *bla*_PSE1 _were not produced in any of the remaining M^A ^isolates. Other research teams have also failed to detect *bla*_OXA1_, *bla*_SHV _and *bla*_PSE1 _in ampicillin-resistant *E. coli *isolates recovered from cattle [20,22]. We are presently in the process of screening for additional β-lactamase determinants in ampicillin-resistant *E. coli i*solates that were not equated with *bla*_TEM1_. A close association of *bla*_TEM1 _with class I integrons has been reported, which likely accounts for the wide dissemination of this determinant among Gram-negative bacteria [53]. Others in Denmark and Spain also found *bla*_TEM1 _to be the most common determinant observed in ampicillin-resistant *E. coli *of animal origin, with *bla*_OXA1 _detected only occasionally [53,54].

## Conclusions

AMR bacteria are clearly able to persist in the bovine gut in the absence of antimicrobial selection pressure, evidenced by ready isolation of tetracycline- and ampicllin-resistant *E. coli *from steers that were not fed antibiotics. This study and previous reports suggest that the occurrence of AMR in commensal *E. coli *harboured by calves is complex, and dependent on multiple factors. Sampling time seemed to affect the presence of certain isolates, which is likely reflecting the transient nature of shedding of specific strains of *E. coli *by cattle. In addition, commonality was higher among isolates obtained from cattle within a pen than between pens, suggesting that animal-to-animal contact plays an important role in the dissemination of AMR bacteria within the feedlot. Feeding a mixture of subtherapeutic antibiotics, in this case tetracycline and sulfamethazine seemed to have a more pronounced impact on the occurrence and persistence of antimicrobial resistance than feeding a single or no antibiotics to cattle. Resistance to SMX and CHL was increased in isolates from the treatment group receiving chlortetracycline and sulfamethazine, which may have arisen from the inclusion of this sulfonamide in the diet. This treatment also appeared to be associated with increased isolation of ampicillin-resistant *E. coli*. Our findings suggest that a more comprehensive understanding of the development and emergence of AMR in feedlots requires that other factors in addition to administration of antimicrobials be taken into consideration.

## Authors' contributions

PM participated in study design and coordination, data analysis and drafted the manuscript. ML and RS contributed to study analysis and experimental techniques. LJY participated in study design and sample collection. ET consulted on environmental implications of transmission of resistance genes. TAM was the overall project leader and participated in design and coordination of project and contributed to the final copy of the manuscript. All authors have read and approve the final manuscript.
